# Factors Influencing Food Choice of Older Black African Adults in the United Kingdom

**DOI:** 10.1177/30495334261424563

**Published:** 2026-02-23

**Authors:** Sophia D. Amenyah, Janet Adjei, Lyndsey Bradley, Sena Yeboah, Charity Agbonisan Aienobe-Asekharen, Hibbah Osei-Kwasi

**Affiliations:** 1 Northumbria University, Newcastle Upon Tyne, UK; 2 Bournemouth University, UK; 3 University of Bristol, UK; 4 Loughborough University, UK

**Keywords:** Photovoice, Older adults, Nutrition, Black African, Ageing, Participatory research

## Abstract

**Background:**

Research shows Black African communities in the UK maintain bicultural dietary patterns combining Westernised and African practices. However, limited research exists on older African adults, who face complex nutrition challenges owing to interacting social and cultural factors affecting health in later life. This study explored factors influencing food choices among older African adults.

**Methods:**

Using Photovoice, an innovative community-based participatory research method, 12 purposively sampled participants were given cameras to photograph their thoughts on eating well and older adults’ health. Semi-structured interviews explored the photos, with thematic analysis conducted on photos and transcripts using an inductive approach.

**Results:**

Participants averaged 62 ± 5.4 years; 75% were female, 58.3% married, 41.7% lived with family, 50% held postgraduate degrees, and 66.7% were fully employed. Key determinants included social, emotional, cultural, age-related health conditions, knowledge, accessibility, nutrition perceptions, creativity, adaptation, technology use, convenience, cost, and time.

**Conclusions:**

This research provides new insights into how older African adults manage the rich, complicated intersection of cultural identity, health needs to support nutrition in ageing. Further research into adaptive strategies, intersectional solutions on culture, health, sociality and technological innovation is warranted to inform culturally tailored age-sensitive interventions for older African adults.

## Introduction

Research indicates a significantly higher burden of non-communicable diseases and nutrition-related long-term conditions in Black African communities, severely impacting quality of life and health in older age (Agyemang et al., 2015; 2. Commission on Race and Ethnic Disparities, 2021; Modesti et al., 2016; Watkinson et al., 2021). After accounting for age, hypertension was highest among Black Caribbean, Black African, and Pakistani adults, whilst women from Black Caribbean (74%), Pakistani (74%), and Black African (73%) backgrounds were most likely to be overweight or obese (NHS Digital, 2022a;NHS Digital, 2022b). Despite the demographic shift in older African adults in the UK and globally, this population remains underserved by research, with limited evidence on their evolving nutritional needs (Bennett et al., 2022; Public Health England, 2017; Robinson, 2018; Wohland et al., 2015). Research on nutritional requirements and policies promoting healthier diets for older adults are neither representative of ethnically diverse populations nor culturally tailored (Mak & Caldeira, 2014; Ruel, 2003) a critical gap identified in the recent SACN report (The Scientific Advisory Committee on Nutrition (SACN; 2021)). Key recommendations include innovative, co-created research investigating the specific nutritional needs of older African adults to enable these communities to meet their needs and shape timely, relevant, tailored policies and interventions improving nutrition and healthy eating in later life.

Individuals from Black African communities (UK-born and migrants) often experience a complex nutrition landscape (Berggreen-Clausen et al., 2022; Harrop, 2016; Karanja et al., 2022; Smeaton et al., 2010) where traditional diets are maintained whilst key features of UK/Western diet and food culture are adopted, presenting complex outcomes for nutrition and health in older age (Bennett et al., 2022; Holmboe-Ottesen & Wandel, 2012; Leung & Stanner, 2011; Ngongalah et al., 2021; H. A. Osei-Kwasi et al., 2016, 2017; Renzaho & Burns, 2006). While limited studies (Asamane et al., 2019; H. A. Osei-Kwasi et al., 2017) have explored these nutritional changes, only few (Asamane et al., 2019, 2020) have focussed on older adults or influenced policy. Given diet’s critical role in healthy ageing, understanding factors driving nutrition inequalities, changing nutritional needs, malnutrition risk, and long-term conditions, while building on cultural influences, is essential. Novel, culturally-tailored research considering cultural, social, and economic contexts is needed to understand this complex landscape and co-develop interventions and policies improving nutrition, healthy ageing, and quality of life among older African adults. This study explored factors influencing food choices among older African adults.

## Methods

### Study design

**Figure 1.**
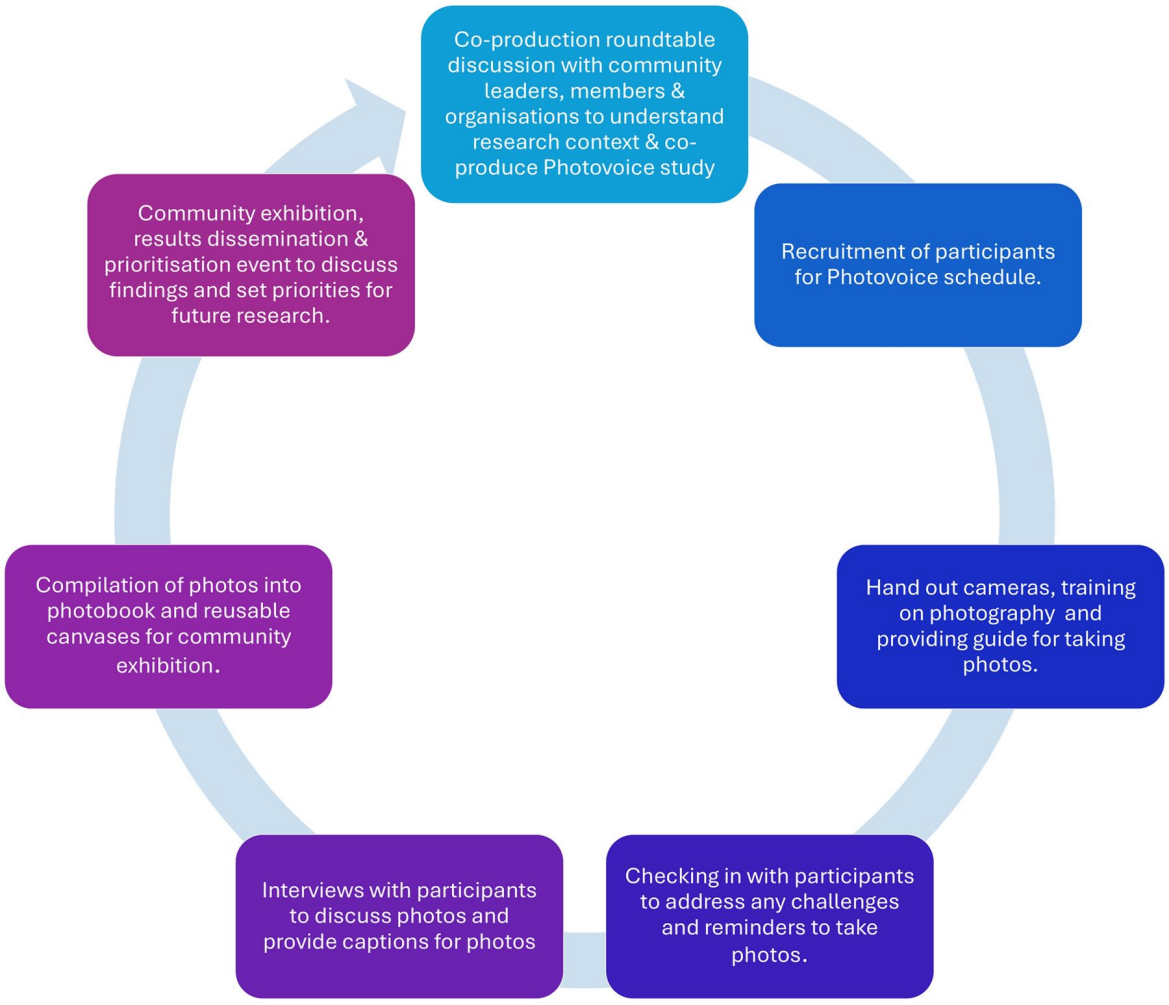
Overview of photovoice study.

A community-based participatory research (CBPR) approach was employed, focussing on working from the ground-up and giving communities a voice on issues, potential solutions, and how to address what matters to them (Suarez-Balcazar et al., 2018, 2020). Consistent with CBPR methods, the study comprised roundtable discussions and a photovoice study (Figure 1). These approaches facilitated equitable co-production and ground-up understanding of nutrition and ageing challenges while co-producing solutions to support healthy ageing. Roundtable discussions are structured, collaborative conversations where participants share ideas and expertise as equals. This method positions all participants as experts, using a decolonising lens to facilitate dialogue and co-created knowledge that resonates with African community practices (Brown & Di Lallo, 2020; Dodge-Francis, 2018; Lavallée, 2009).

### Roundtable discussions

Two virtual roundtable discussions were organised to co-produce the research and assess its relevance to community needs. Participants from across the UK were recruited through community organisations and personal contacts of the lead researchers. Each roundtable comprised 10 participants, including older adults, middle-aged individuals, and men and women who self-identified as Black African. The discussions, facilitated by SDA and HOK, lasted approximately 60 to 90 min (Supplemental Table 1).

### Photovoice

Following the round table discussions, a photovoice study was conducted. Participants included older adults from Black African communities (both born in the UK and migrants) across England and Wales. Photovoice is a participatory photography and digital storytelling method which provides an accessible way to describe realities, perspectives and raise awareness (Budig et al., 2018; Wang & Burris, 1997). It provides an alternative to traditional qualitative methods and is suited for use with populations who have been disenfranchised by traditional research methods (K. Anderson et al., 2023; Budig et al., 2018; Conde et al., 2024). Photovoice has been used to study a wide range of topics in older adults, ranging from health issues and the impact of the environment on health and wellbeing (Heinz et al., 2023; Ottoni et al., 2023). The use of Photovoice to document nutrition and ageing in older African adults in the UK is a novel aspect of this study.

### Participants and recruitment

Purposive sampling was used to recruit 15 participants, sample size consistent with qualitative literature (Hennink & Kaiser, 2022) from faith-based organisations and Black African community groups using information sheets, mobile phone calls, social media, word-of-mouth, and snowballing techniques. Additionally, flyers were advertised at international food shops, hairdressers, and on social media, following previous experience working with this community (Amenyah et al., 2025).

Participants were eligible if aged 55 years and above and self-identified as Black African. Consistent with NIHR INCLUDE guidelines (Treweek et al., 2021) on including underserved communities in research, no other exclusion criteria were applied; individuals with capacity to consent and participate were eligible. The study was conducted in accordance with the Declaration of Helsinki, and informed consent was obtained before enrolment, with additional verbal consent obtained before data collection. Participants were assured of confidentiality and anonymity before interviews. All data were anonymised, and participants were assigned unique study identification numbers to ensure confidentiality and prevent inclusion of identifying characteristics in manuscripts, reports, and publications.

### Data collection

Data collection followed the adaptation of steps outlined in Wang and Burris (1997) and H. Osei-Kwasi et al. (2023). The first step involved participant consent and photography training. Photography ethics, including anonymity protocols for identifying individuals, were also discussed. Participants were then asked to take photographs using a pre-specified brief (Supplemental Table 2). In-depth interviews using the SHOWeD technique (Wang & Burris, 1997) were conducted in English to discuss selected photographs in detail, revealing nuanced insights and meanings related to nutrition and ageing. All interview transcripts were transcribed verbatim for analysis.

### Data synthesis and analysis

Data, including photos and transcripts of semi-quantitative interviews, were analysed using NVivo Pro 12.5 (QSR International, 2020), which allowed the coding of photos as well as text. Thematic analysis (Braun & Clarke, 2006, 2023) was conducted using an inductive approach (Thomas, 2006). The inductive approach allowed key themes to emerge naturally from the raw data, focussing on those that were most frequent, dominant, or significant. The analysis was primarily undertaken using the iterative six-phase process outlined by Braun and Clarke (Braun & Clarke, 2006) to ensure methodological rigour. The familiarisation phase involved reviewing audio recordings, transcripts, field notes, and photographs to identify key ideas. All coders agreed on a codebook applied to all transcripts in NVivo. This ensured consistency across researchers. NVivo was used for line-by-line coding to develop themes, with discrepancies discussed among all coders. A photo exhibition and photobook were curated to showcase outputs from the research.

## Results

Socio-demographic characteristics are presented in Table 1. Of 15 recruited participants, 12 completed the study (80% completion rate). Participants included nine females (75%) and three males (25%), with a mean age of 62 years across seven UK locations. Eleven participants had migrated to the UK, with one UK-born. Half held postgraduate degrees (*n* = 6), and two-thirds were in full-time employment (*n* = 8). Annual household income varied widely: 33.3% (*n* = 4) earned under £20,000, while 33.3% (*n* = 4) earned over £80,000. Most participants were Christian (75%, *n* = 9) and lived with family, including extended family members (41.7%, *n* = 5).

**Table 1.** Demographic Characteristics of Participants (n = 12).

Variable	*n* (%), Mean (*SD*)
Sex	
Male	3 (25.0)
Female	9 (75.0)
Age	62 (5.4)
Marital status	
Married	7 (58.3)
Separated/divorced	2 (16.7)
Widowed	2 (16.7)
Single	1 (8.3)
Education	
No formal education	1 (8.3)
Compulsory schooling	2 (16.7)
Undergraduate degree	3 (25.0)
Postgraduate degree	6 (50.0)
Employment	
Full time employed	8 (66.7)
Unemployed	2 (16.7)
Retired	2 (16.7)
Income	
Less than £20000	4 (33.3)
£20,000- £39,999	2 (16.7)
£40,000-£59,999	0 (0.0)
£60,000-£80,000	2 (16.7)
Above £80,000	4 (33.3)
Faith/religion	
Christian	9 (75.0)
Muslim	3 (25.0)
Household	
Living alone	2 (16.7)
Living with partner and children	4 (33.3)
Living with family, including extended family members	5 (41.7)
Living with friends and acquaintances	1 (8.3)
Country of birth	
Gambia	1 (9.1)
Ghana	7 (63.6)
Nigeria	3 (25.0)
UK	1 (9.1)
UK location	
Southwest	2 (16.7)
Southeast	3 (25.0)
Northwest	3 (25.0)
South	1 (8.3)
East of England	1 (8.3)
London	1 (8.3)
Wales	1 (8.3)

*Note*. Data expressed as mean (standard deviation) for continuous variables and frequency (%) for categorical variables.

### Emerging themes

**Figure 2.**
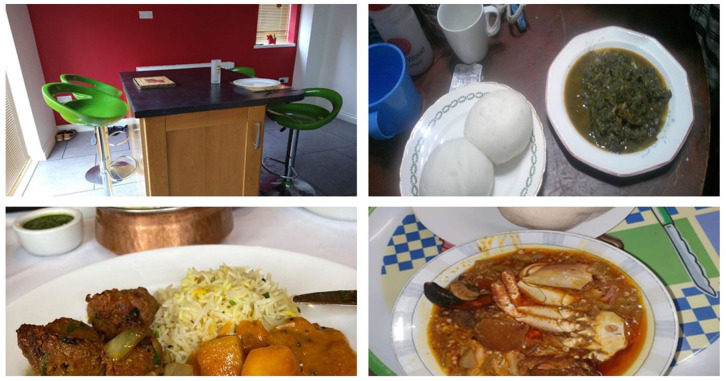
Participants’ photos illustrating the theme of social, emotional, and cultural determinants.

**Figure 3.**
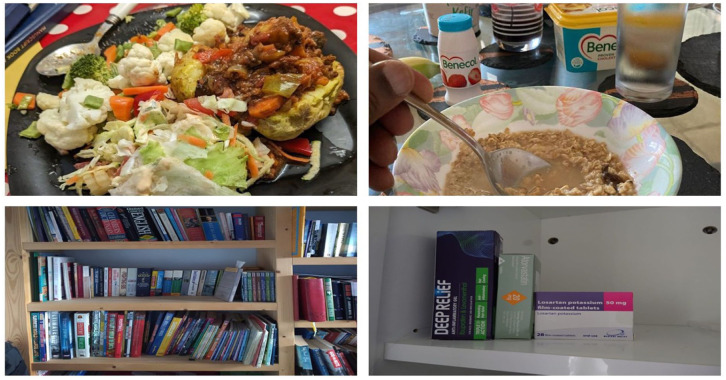
Participants’ photos illustrating the theme of age and health conditions.

**Figure 4.**
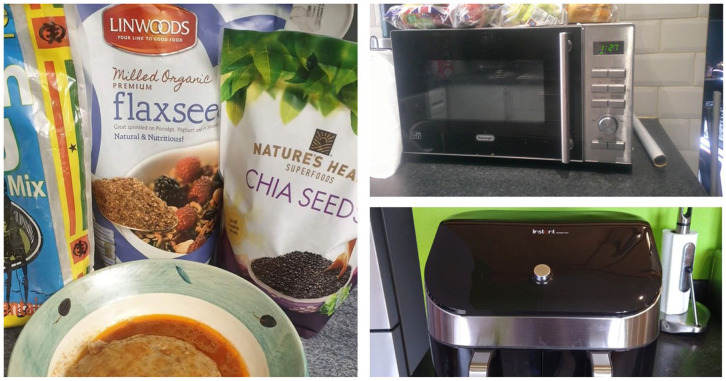
Participants’ photos illustrating the themes around knowledge, accessibility, and perceptions around nutrition, creativity, adaptation & using technology.

**Figure 5.**
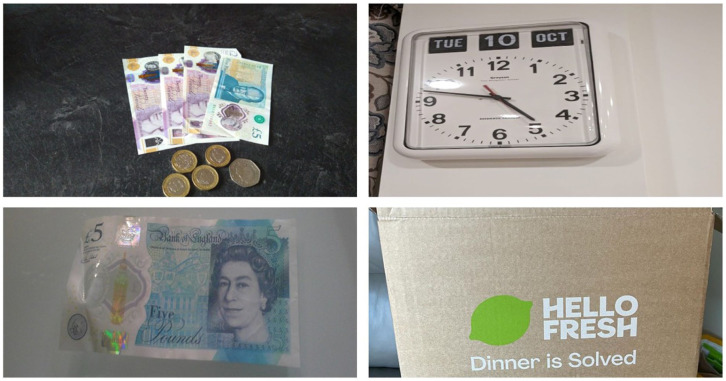
Participants’ photos illustrating the theme on convenience, cost and time.

The inductive thematic analysis identified five primary themes: Social, emotional, and cultural determinants; Age and health conditions; Knowledge, accessibility, and perceptions around nutrition; Convenience, cost and time; Creativity, adaptation & using technology (Figures 2–5).

### Theme 1: Social, emotional, and cultural determinants

Cultural influences emerged as a predominant factor underlying participants’ food choices, with social and emotional considerations playing significant roles in their evolving dietary decisions. This was particularly evident in participants’ strong attachment to traditional cuisine, which made them feel closer to their heritage and facilitated joyful reminiscing. For example, participants discussed ‘homemade meals’ that enabled them to better connect with their culture and home in Africa.

*‘. . . .for older people, they lavish this food, they love this food, because it reminds them of home. . . . .there is this Waakye too. Because when I get a food, and when I eat this food, it reminds me of home. And again, when I eat it, I feel, full of like nostalgia. I don’t cook Waakye every time, it’s a food I cook maybe once every three months, or one every two months, because the boys don’t like it, but when I cook it, I feel, it makes me feel home’* (F4YO22).

A key aspect of culture as a driver of food choice was a strong link to tradition and the importance of passing down traditions to younger generations.

*‘You cannot throw away your, you know, you use all your young days. You know, we pass it on, we talk about it, and the children will always let you cook, and actually, African children, always like the African native food, so, you have to teach them how to make a pounded yam, from scratch’ (F4Y023)*.

#### Loneliness and family influence

While food served as a symbol of joyful reminiscence, it also indicated loneliness. Participants revealed that social isolation significantly influenced their food choices, nutrition, and behaviours, particularly meal preparation and food consumed. Several participants indicated that motivation to prepare home-cooked meals diminished considerably when dining alone, often resulting in a preference for convenient options such as takeaways.

*‘You see as soon as the children left home, sometimes my husband is also away, I find that when I’m on my own I don’t feel like getting up to go and cook a whole meal. So, I think that if for a long period I was on my own I would be tempted to take the shortcut, or the easier way out and not eat as well as I should. Because I know that on the odd day, I do that when I’m the only one left. ‘And then you just have to settle for maybe takeaways, or you know ready meals.’ (F4Y024)*.

Participants described how their dietary patterns evolved as their household composition changed, particularly when adult children left home. This transition period appeared to mark a significant shift in their food choices and meal preparation habits:

*‘One factor may be loneliness, because children have moved away, or yeah, you’re on your own’ (F4Y021)*.

#### Cultural influence and traditional practices

Although food choices were connected to loneliness, food also bridged their African heritage and new life in the UK. Participants revealed that cultural heritage from Africa significantly shaped their current dietary choices, with many expressing strong adherence to traditional African dietary practices. This cultural influence was particularly evident in their perceptions of how food should be sourced and prepared.

*‘As Africans, okay, we are used to doing Banku with all this oil on; we have that mentality, if there is oil, it means your food is rich’ (F4Y022).*

Interestingly, whilst cultural identity remained a strong influence on food choices, some participants described how newly acquired nutritional knowledge in the UK prompted modifications to traditional staple foods. Participants connected their ethnicity to food when discussing these knowledge-driven dietary changes, which were expressed through addition, reduction, or substitution. Addition involved adding chia seeds, flax seeds, or more vegetables; reduction involved decreasing oil and portion sizes; and substitution involved replacing palm oil and traditional fufu with olive oil and oats, or other healthier options.

*‘But instead of using a lot of palm oil, I’ll cook with healthier oil first, like olive oil, and then just put a little bit, maybe a tablespoon of palm oil, just to get that authentic Ghanaian flavour with palm oil. So, whereas I mean before I came here, I would never have dreamt of using olive oil. And now that I know that look, palm oil isn’t as healthy, then you know I’ve switched to a healthier oil’ (F4Y024)*.

### Theme 2: Age and health conditions

Age-related considerations and health conditions strongly influenced participants’ food choices, prompting culinary changes. Participants described implementing specific, often untraditional dietary modifications in response to health diagnoses, whilst others adopted preventive measures due to family health histories. These health conditions were sometimes observed as family trends, prompting culinary changes that deviated from usual practices. Such changes typically involved addition, reduction, or substitution of certain foods; for instance, incorporating more vegetables, reducing oil quantity, or replacing traditional fufu with oats.

‘*You see that there’s a lot of vegetables on the plate. And I’m not faking it. It’s something that I’m doing now. It’s something because I come from a family where there’s diabetes. And so I went for the check up and the result was that I was pre-diabetic*’ *(F4Y003).*

#### Age-related dietary changes

Participants demonstrated clear recognition of the relationship between ageing and dietary needs, acknowledging the increasing importance of appropriate nutritional choices in later life. Participants were aware they needed to make new, consistent dietary changes that would yield positive outcomes over time, particularly to promote health and prolong life.

*‘. . . . . . .some people think that as you get older you don’t need much food. Or you don’t need to eat protein and things. Anything small will be okay. And I think that’s a misconception, because we do need to have, we do need you know all the nutrients in terms of a healthy diet. We may not need the quantities we did when we were growing, but we still need to eat well, and healthily’ (F4Y024)*.

#### Chronic disease management

Despite their love for cultural foods, participants were aware they had to make drastic dietary changes if they had certain health conditions. For instance, participants with existing health conditions exhibited heightened awareness of dietary implications and actively modified their food choices to manage their conditions. These new choices were sometimes indirectly challenged by close relationships and the desire to eat previously enjoyed foods.

*‘Because I am aware that, you know I have, I am at risk of high cholesterol and high blood pressure, so, then, I don’t want to eat too much of the fatty and the salty things’ (F4Y024)*.

*‘Not now. Yes, it used to be like that. It used to be like that, but now, not necessarily, Because I have to an age and a point that I think I need to think about me, especially when they told me [about medical condition], so, I’m, and even with them [family members], I encourage them, like the pasta, I won’t eat it, I cooked it for them, I don’t even eat it, but that was the quickest food for them. So, in the past, yes, it was influenced what I ate, but not anymore, because now I’m thinking about me and my health, to keep me healthier’ (F4Y022)*.

### Theme 3: Knowledge, accessibility, and perceptions around nutrition

Participants shared how their food choices were significantly influenced by their nutritional knowledge, food accessibility, and their perceptions of healthy food. Some participants noted how their traditional dietary practices were being modified through enhanced nutritional understanding and availability.

*‘I mean it’s true that generally, we will eat the foods that we are used to in Ghana. But it’s modified based on the knowledge we now have’ (F4Y024)*.

#### Nutrition knowledge, literacy and trust

Participants’ culinary decisions were influenced by their awareness of specific health benefits associated with certain foods, motivating regular consumption of particular vegetables. However, limited trust in food products also shaped their choices. Participants managing health conditions were unwilling to rely on products with uncertain contents, often going to extra lengths such as grinding oats for fufu to ensure they could trust what they consumed.

*‘This is Fufu made, which I added like flax seed and chia seed and Plantain Fufu powder and there’s a bit of oat as well, organic rolled oats’ (F4Y022)*.

#### Health information sources

The study revealed that participants accessed health information through diverse channels, including interpersonal communication, media sources, and independent research. These information sources subsequently informed their nutritional decisions, as demonstrated by participants’ accounts of evidence-based dietary choices.

*‘And in Benecol, apparently, the drink has been proven to lower cholesterol, and I think there is some evidence of that, so, I drink a tiny bottle of Benecol a day. And then I also know, I introduced it to a friend of ours, who had high cholesterol’ (F4Y021)*.

### Theme 4: Creativity, adaptation & using technology

Participants shared different approaches and creative strategies for maintaining healthy eating habits, particularly in adapting traditional foods. This was usually geared towards maintaining a healthy lifestyle while still enjoying traditional foods they loved. They demonstrated innovative approaches to modifying traditional recipes to enhance nutritional value. To facilitate healthier cooking practices and maintain their health, participants utilised various technological interventions, from mobile applications to cooking appliances like air fryers and ovens. These practices were often informed by the diverse health information channels they relied on.

*‘The ‘towel’, yeah yeah, the intestine; Yeah, that one too, I will wash it and and, and put it in the oven. So that the fat comes out of it. And so, if you use it for light soup, for example, you hardly see any, any oil on top of the soup. Because most of the oil comes out and so I’m able to have a bit of all that without feeling, any guilt or feeling bad. That I’m killing myself because I believe I’ve, I’ve treated it to a point where it safe too. It’s quite safe to eat’ (F4Y003)*.

### Theme 5: Convenience, cost and time

Time and convenience strongly influenced participants’ food choices as they pursued healthy lifestyles. These determinants were discussed in relation to ageing and financial resources, with better financial positioning facilitating healthier ageing. Convenience and time constraints posed universal challenges, particularly for employed participants, affecting their capacity to prepare nutritious meals. Some solutions included ordering pre-prepared meal boxes; however, these were associated with higher costs.

*‘Busy lives, busy at work, rushing, leaving home, you come to work, it’s late, and so, time is always a limiting factor. And so, the danger is, well, find something very easy, and necessarily be all that nutritious, but yeah, you fill your tummy and there you go. And so, you can be, time can always be the taskmaster on your back all the time, and you go for the easiest option, alright, let’s call the takeaway, because, yeah, I’m tired, I can’t be bothered to cook, so, order a takeaway, and we know, yeah, if you do it sparingly it’s not a problem, but if because of time, you end up having to takeaways so many times a week, and that is not healthy, it is not a healthy route’ (F4Y021)*.

Some participants shared how time pressures often resulted in selecting more convenient but less varied food options. This was significant as some participants perceived food variety as signalling a healthy meal, with variety indicating ‘healthy’, ‘colourful’, and greater nutritional value.

*‘Time is a factor because it limits the clock, time limits the variety I can eat’ (F4Y003)*.

*‘If I was on pension and at home, I am sure would have eaten more healthy food; but when you are old and you are still working, you are not able to eat healthy food more frequently’ (F4Y004)*.

As time and convenience were associated with some level of cost, financial considerations also emerged as a major barrier to healthy eating for most participants. This made the price of food items a key factor as participants had to weigh their finances against the benefit of healthy eating.

*‘You know, sometimes I go to the shop, I cannot buy it because the money they. You know, the money is cost. I can’t afford it’ (F4Y009)*.

However, participants also demonstrated a positive cost-benefit analysis approach, shown by prioritising health benefits over financial constraints. This was prominent among participants who had good nutritional knowledge, information from various sources, and who had to manage a medical condition.

*‘So, then it’s more expensive for me, but I look at my health, I look at the knowledge, I look at everything, and I’ll take it like that, you know’ (F4Y024)*.

## Discussion

This study provides the first evidence using Photovoice to examine factors influencing nutrition and ageing in older African adults. It reveals a more complex picture than simple binary acculturation models suggest, showing that older African adults selectively adapt traditional foods based on their knowledge rather than blanket adoption of Western diets. Importantly, social isolation significantly influenced what they eat, a social-structural dimension, often missing from nutrition research that focuses primarily on individual knowledge or cultural preferences. The findings indicate a complex cost-benefit analysis that extends beyond simple affordability and captures both the positive and negative aspects of cultural food attachments. The findings align with previous research in younger African populations identifying tradition, culture, costs, and family influence as key drivers of food choice and dietary practices (C. A. Anderson et al., 2019; Horlyck-Romanovsky et al., 2021; H. A. Osei-Kwasi et al., 2017). A recent study of intergenerational dietary acculturation among Ghanaian immigrants in the US showed adults maintaining cultural traditions whilst facilitating dietary acculturation for youth, with both traditional and global diets evolving as families adopted new foods and healthy social norms (Horlyck-Romanovsky et al., 2021). Similarly, Ojo and colleagues’ systematic review and qualitative study (Ojo et al., 2023a; 2023b) identified eating patterns, social and cultural factors, food preferences and routines, accessibility and availability, health and healthy eating, and perceptions of UK government healthy eating resources as factors that significantly influenced dietary choices among African populations in the UK.

In contrast to previous studies, social isolation, health conditions, and managing age-related health emerged as key drivers for healthier food choices, reflecting older adults’ motivations and factors influencing their perceptions of ageing. Participants were more likely to engage in self-led learning following a medical diagnosis, linked closely to gaining knowledge for healthy eating and ageing. Knowledge influenced new approaches to traditional culinary practices, perceptions of healthy and unhealthy meals, and facilitated acceptance of new technologies. A key finding was participants’ reliance on trusted knowledge sources, providing opportunities for culturally tailored interventions and policies supporting nutrition and ageing in older African adults.

Interestingly, participants demonstrated innovative strategies to adapt to their food environments and expressed a strong interest in using technology to enhance convenience and facilitate healthier eating. Meal boxes, for instance, offered practical solutions for cooking in smaller households, a key challenge for older adults (Björnwall et al., 2021; Gardiner et al., 2018; Suthutvoravut et al., 2019), while addressing convenience and time constraints. However, higher costs made this approach predominantly accessible to higher-income participants.

Altogether, this research identifies different factors influencing nutrition and ageing in older African adults and demonstrates how these interact with one another to shape dietary practices. Future interventions should prioritise promoting and improving accessibility of traditional food substitutes, alongside developing novel approaches to cultural foods that support healthy ageing whilst preserving cultural identity. Encouraging kitchen technologies that emulate traditional preparation methods may reconcile tensions between modern convenience and cultural authenticity. When properly applied, technology and innovation can serve to support cultural preservation rather than undermine it. Effective interventions should be grounded in existing adaptive strategies rather than importing external solutions, recognising that cultural preservation of food and health optimisation are not conflicting goals. Facilitating conditions where older adults can make healthy food choices without restrictions of convenience, cost, or availability may empower more intuitive and sustainable dietary decision-making. Ultimately, nutrition interventions require intersectional solutions that address the numerous interacting determinants of health and wellbeing within communities.

A key strength of this study is its use of Photovoice, a CBPR method, to explore factors influencing nutrition and ageing among older African adults, a group currently underserved by research. This approach enabled participants’ and community voices to guide the inquiry, highlighting and challenging common assumptions, including resistance to technology, incompatibility between traditional diets and health guidelines, and the need to avoid innovation to preserve culture, and provides evidence to inform interventions that support healthy ageing. Using a small sample size, it captured data from diverse participants across income groups and successfully demonstrated the feasibility of Photovoice for co-producing research with older African adults. However, most participants were female, highly educated, and in full-time employment. Future studies considering intersectionality are warranted to reach wider groups of older African adults and to co-produce inclusive interventions. Additionally, due to the inductive nature of our analysis, other factors that may influence nutrition and ageing in this demographic were not captured and should be explored in future research.

## Conclusions

This study provides new insights and highlights significant determinants of nutrition and healthy ageing among older African adults. The findings provide the foundation for future research with older African adults using participatory methods. Additionally, it underscores the need for further investigation into the complex interactions between nutritional knowledge, cultural traditions, technology and social factors, including how these are weighted and valued to inform the development of effective, culturally tailored, community-based nutrition interventions for this population.

## Supplemental Material

sj-docx-1-ggm-10.1177_30495334261424563 – Supplemental material for Factors Influencing Food Choice of Older Black African Adults in the United Kingdom Supplemental material, sj-docx-1-ggm-10.1177_30495334261424563 for Factors Influencing Food Choice of Older Black African Adults in the United Kingdom by Sophia D. Amenyah, Janet Adjei, Lyndsey Bradley, Sena Yeboah, Charity Agbonisan Aienobe-Asekharen and Hibbah Osei-Kwasi in Sage Open Aging
