# Supplementary material for: Factors Influencing Food Choice of Older Black African Adults in the United Kingdom
Source: Sage Open Aging. 2026 Feb 23;12:30495334261424563. doi: 10.1177/30495334261424563 (PMC12929829; doi:10.1177/30495334261424563)
Supplement: sj-docx-1-ggm-10.1177_30495334261424563 – Supplemental material for Factors Influencing Food Choice of Older Black African Adults in the United Kingdom [file sj-docx-1-ggm-10.1177_30495334261424563.docx]

**Supplementary data**

**Supplementary table 1:** Roundtable discussion questions

| 1. What types of challenges do older people from the African community face in meeting their nutritional requirements? 2. What are the factors that drive these challenges? 3. How would we describe the current food landscape for older people from the African community? 4. What are the solutions to the challenges we have outlined? 5. How can we implement the solutions identified? 6. Who needs to be involved in implementing the solutions and how can we reach them? |
| --- |

**Supplementary table 2:** Photovoice photo taking brief

| 1. A photo of what you consider as healthy food. 2. A photo of what you consider as unhealthy food. 3. A photo showing any changes made to what and how you eat since living in the UK. 4. A photo describing the biggest factor that affects how and what you eat. 5. A photo that describes the things that make eating well difficult as one grows older. 6. A photo that describes the things that make eating well easy as one grows older. 7. A photo that describes what you think of you when you think of eating well and people’s health when they are older. |
| --- |
